# A revision of the distribution of sea kraits (Reptilia, *Laticauda*) with an updated occurrence dataset for ecological and conservation research

**DOI:** 10.3897/zookeys.569.6975

**Published:** 2016-02-26

**Authors:** Iulian Gherghel, Monica Papeş, François Brischoux, Tiberiu Sahlean, Alexandru Strugariu

**Affiliations:** 1 Department of Integrative Biology, Oklahoma State University, 501 Life Sciences West, Stillwater 74078, Oklahoma, USA; 2 Current address: Department of Biology, Case Western Reserve University, 2080 Adelbert Road, Cleveland 44106, Ohio USA; 3 Centre d’Etudes Biologiques de Chizé, CEBC-CNRS UMR 7372, 79360 Villiers en Bois, France; 4 Faculty of Biology, University of Bucharest, Bvd. Mihail Kogalniceanu no. 36-46 Bucharest, Romania; 5 Department of Terrestrial Fauna, “Grigore Antipa” National Museum of Natural History, Kiseleff no 1, 011341, Bucharest, Romania; 6 Faculty of Biology, “Alexandru Ioan Cuza” University of Iași, 20th Carol 1st Boulevard, 700505, Iasi, Romania

**Keywords:** Amphibious snakes, Elapidae, geodatabase, distribution, marine, open access, sea snakes

## Abstract

The genus *Laticauda* (Reptilia: Elapidae), commonly known as sea kraits, comprises eight species of marine amphibious snakes distributed along the shores of the Western Pacific Ocean and the Eastern Indian Ocean. We review the information available on the geographic range of sea kraits and analyze their distribution patterns. Generally, we found that south and south-west of Japan, Philippines Archipelago, parts of Indonesia, and Vanuatu have the highest diversity of sea krait species. Further, we compiled the information available on sea kraits’ occurrences from a variety of sources, including museum records, field surveys, and the scientific literature. The final database comprises 694 occurrence records, with *Laticauda colubrina* having the highest number of records and *Laticauda schistorhyncha* the lowest. The occurrence records were georeferenced and compiled as a database for each sea krait species. This database can be freely used for future studies.

## Introduction

Sea kraits (genus *Laticauda*) are a group of amphibious, marine snakes from the family Elapidae (Heatwole et al. 2005; Pyron et al. 2011; Shine et al. 2002, 2003), distributed in tropical and subtropical coastal waters of the eastern Indian Ocean, south-east Asia, and archipelagoes of the western Pacific Ocean (Heatwole et al. 2005).

The taxonomic status of the group has been and continues to be subject to much debate. For instance, Vitt and Caldwell (2009) recently elevated Laticaudinae as a separate subfamily within elapids. In contrast, Kharin and Czeblukov (2013) elevated the subfamily Laticaudinae to familial level (Laticaudidae) and divided the genus *Laticauda* into two genera (*Laticauda* and *Pseudolaticauda*), based on morphological characters. However, this split is not widely accepted (e.g., Elfes et al. 2013) and the sea kraits are considered to belong to a single genus, *Laticauda*. Furthermore, in a phylogenetic analysis of the group, Pyron et al. (2011) did not recognize any subfamilies within elapids, as none of the previously described ones (Elapinae, Hydrophiinae and Laticaudinae) formed well-supported monophyletic groups.

In contrast with the taxonomic disputes, from an ecological perspective, this clade is divided into three major complexes (Heatwole et al. 2005), which broadly overlap in geographic range, but differ in their relative use of terrestrial versus marine environments. Species from the “*Laticauda colubrina* complex” (Yellow-banded sea kraits, composed of *Laticauda colubrina*, *Laticauda frontalis*, *Laticauda guineai*, and *Laticauda saintgironsi*) are more terrestrial; species from the “*Laticauda semifasciata* complex” (Black-banded sea kraits, composed of *Laticauda semifasciata* and *Laticauda schistorhyncha*) are more aquatic; and species from the “*Laticauda laticaudata* complex” (Blue-banded sea kraits, composed of *Laticauda laticaudata* and *Laticauda crockeri*) are considered intermediate (Greer 1997; Heatwole 1999, Brischoux et al. 2013).

As amphibious animals, sea kraits have unique characteristics that allow them to perform well in both marine and terrestrial environments. For instance, as sea snakes, they display a paddle-shaped tail that allows them to move efficiently in the water (Brischoux et al. 2010; Brischoux and Shine 2011), but have retained terrestrial characteristics such as large ventral scales that allow them to crawl efficiently on land (Bonnet et al. 2005; Shine and Shetty 2001). Although they prey mostly on eels in coral reefs, sea kraits need to return on land (to digest, rest, slough their skin, mate, and lay eggs; Heatwole 1999) where they manifest a high degree of philopatry (Brischoux et al. 2009; Brischoux et al. 2007; Shetty and Shine 2002).

Interestingly, it has been recently shown that acquisition of fresh water is crucial for sea kraits (Kidera et al. 2013; Lillywhite et al. 2008) and that a combination of availability of fresh water on land and low oceanic salinity at sea may determine environmental tolerances and geographic distributions of sea kraits (Brischoux et al. 2012, 2013). Also, studies indicate that sea snakes may act as indicators of the effects of climate change (Lillywhite et al. 2008, 2014; Lillywhite and Tu 2011), and there is growing interest in their conservation (Bonnet 2012; Bonnet et al. 2009; Brischoux et al. 2009; Elfes et al. 2013).

As such, detailed knowledge regarding the distribution of sea kraits is key for applying conservation measures, planning conservation reserves, and evaluating the impact of human activities (Elith et al. 2006; Ferrier and Watson 1997; Funk and Richardson 2002; Rushton et al. 2004). In the present study, we review the information available on the geographic range of the three sea krait groups and analyze their distribution patterns. In addition, we provide an occurrence database for each sea krait species for use in future studies.

## Materials and methods

### Occurrence records

A database of sea krait occurrences was created using a combination of data extracted from online repositories (GBIF, HerpNet, iOBIS), from published scientific literature, and from field surveys. Most of the occurrences came from the marine environment, as data on terrestrial localities are scarce for this group. Because of philopatry, sea kraits generally avoid venturing very far from the shore line (Lane and Shine 2011a). Occurrences without spatial data were manually georeferenced to the finest scale possible using the information provided by the source and Google Earth 7. Country taxa lists or locations that could not be georeferenced due to lack of detailed locality descriptions (e.g., name of islands or provinces within countries; McCarty 1986; David and Ineich 1999) had to be excluded from our dataset, as the descriptions were too general. The resolution of the final dataset is 9 km and was projected to WGS84. This resolution is standardized in accordance with existing environmental data (e.g., Bio-ORACLE, www.oracle.ugent.be/) that can be used to answer various biogeographic, conservation or evolutionary questions.

### Distribution patterns of sea kraits

Distribution maps for all species of sea kraits were created in ArcGIS 10.2 (ESRI 2011) by intersecting the occurrence points with the 100 km Military Grid System (MGRS) available on-line from the National Geospatial-Intelligence Agency (NGA 2014).

The Extent of Occurrence (EOO) and the Area of Occupancy (AOO) were calculated according to the methodology proposed by IUCN (2012) using ArcGIS 10.2 (ESRI 2011). The calculations for EOO were based on the occurrence records gathered, while the AOO also relied on the 100 km MGRS. In our study, the AOO is overestimated because of the 100 km MGRS used for measurements.

An optimized hot spot analysis was performed using the occurrence points for all species of sea kraits and the whole grid in order to identify statistically significant clusters of high values (hot spots) or low values (cold spots) (ESRI 2011). Subsequently, the analysis was re-run using all occurrence points for the whole *Laticauda* group, but the area was limited to those cells that contained at least one occurrence. The aim was to detect areas in the distribution range of sea kraits that can be viewed as hot spots for the group (i.e., areas with high clustering of species).

Finally, the Shannon-Wiener diversity index was calculated for the group based on the number of occurrences in each cell of the grid, using the Marine Geospatial Ecology Tools (MGET) toolbox (Roberts et al. 2010). The classification scheme used for the index was based on the Natural Breaks (Jenks) algorithm (ESRI 2014).

## Results

The final database was comprised of 694 unique records of occurrence at a spatial resolution of 9 km (Suppl. material 1). The bulk of these records belonged to the yellow-banded sea krait (*Laticauda colubrina*, 64.55% of all records compiled), while the lowest number of occurrences was for *Laticauda schistorhynchus* (0.86% of all records) (Table 1).

**Table 1.** Number of occurrence records available for each species in the *Laticauda* group.

Species	Number of occurrence records	% of total no. of occurrences
*Laticauda colubrina*	448	64.55
*Laticauda frontalis*	18	2.6
*Laticauda guineai*	10	1.44
*Laticauda laticaudata*	108	15.56
*Laticauda saintgironsi*	75	10.81
*Laticauda schistorhynchus*	6	0.86
*Laticauda semifasciata*	29	4.18

The EOO registered very high values for *Laticauda colubrina* as a result of its wide distribution range, while *Laticauda schistorhynchus* had the smallest EOO, with only 180.99km^2^ (Table 2). The AOO was also the smallest for *Laticauda schistorhynchus*, while the largest AOOs were for *Laticauda colubrina* and *Laticauda laticaudata* (Table 3).

**Figure 1.**
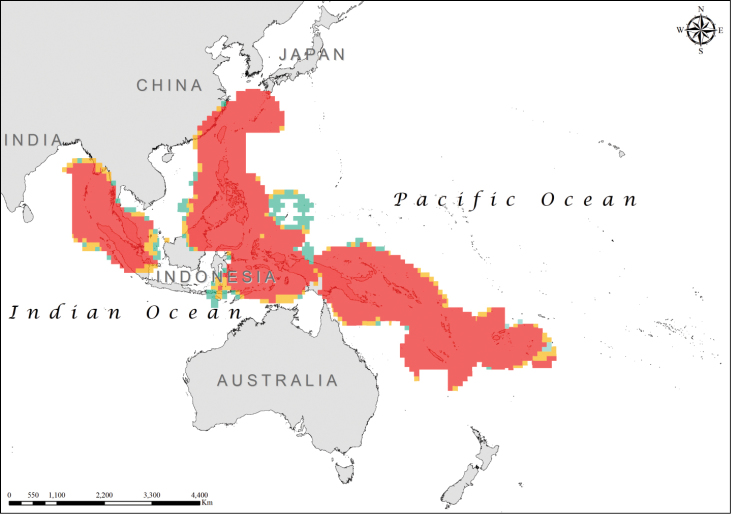
Distribution of hot spots for the *Laticauda* group (color codes reflect statistical confidence; red for 99% confidence level, orange for 95% confidence level, and green for 90% confidence level).

The optimized hot spot analysis based on the whole MGRS grid identified the bulk of the range (western shores of Myanmar and Thailand, Indonesia, Malaysia, Philippines, Papua New Guinea, Solomon Islands, Vanuatu, and Fiji) as an area of high spatial clustering, with 99% confidence (*z* = 9.015; *p* = <0.001), while areas based on 95% confidence (*z* = 3.21; p = 0.001) generally omitted them (Figure 1). Important regions of high spatial clustering, but with a reduced degree of confidence (90%) (*z* = 2.84; *p* = 0.004), were located around the island of Palau and to the north of Papua New Guinea (Figure 1).

**Table 2.** Extent of Occurrence (EOO) for the species of *Laticauda* group.

Species	Extent of occurrence (sq. km.)	Of which	
		% Land	% Ocean
*Laticauda colubrina*	31,651,270.32	18.43	81.57
*Laticauda frontalis*	93,874.79	7.99	92.01
*Laticauda guineai*	6,461.75	83.27	16.73
*Laticauda laticaudata*	27,350,493.24	15.74	84.26
*Laticauda saintgironsi*	87,825.41	24.19	75.81
*Laticauda schistorhynchus*	180.99	83.68	16.32
*Laticauda semifasciata*	6,006,752.15	15.50	84.50

**Figure 2.**
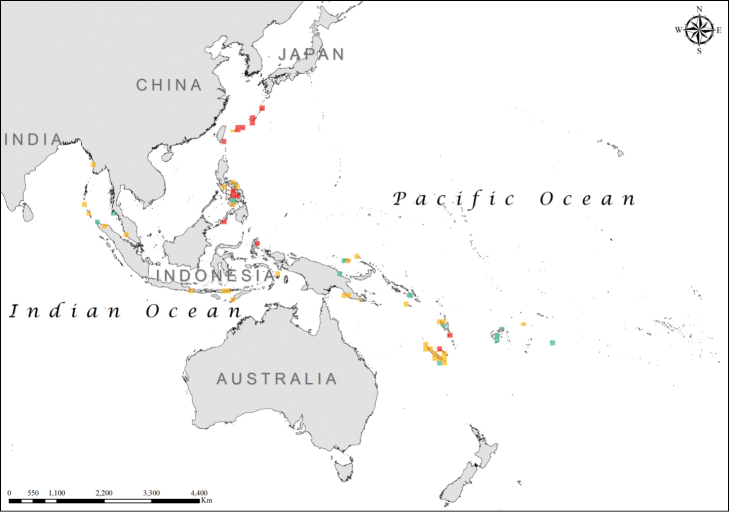
Shannon-Wiener diversity index for the *Laticauda* group (green: *H*’ = 0.000001–0.56; orange: *H*’ = 0.57–0.82; red: *H*’ = 0.82–1.08).

The Shannon–Wiener diversity index registered values between 0 and 1.089 and the diversity map created (Figure 2) showed high diversity values (>0.82) for sea kraits on the islands south-west of Japan and south-east of Taiwan, in the Visayan Sea from the Philippines Archipelago, in the northern part of Celebes Sea, on the northern shores of Halmahera, Indonesia, in New Caledonia around the atoll Ouvéa, and in the Coral Sea around the island of Efate, Republic of Vanuatu (Figure 2).

## Discussion

Through the current study we provide the first set of comprehensive distribution maps for all exant species of sea kraits *Laticauda* spp. (but see also The IUCN Red List online maps). Following the pertinent suggestions of Heatwole and Cogger (2013), cases of vagrancy were excluded from the current analysis. Complementary analyses regarding the conservation and distribution of marine elapid snakes (including sea kraits) have been published by Elfes et al. (2013).

**Figure 3.**
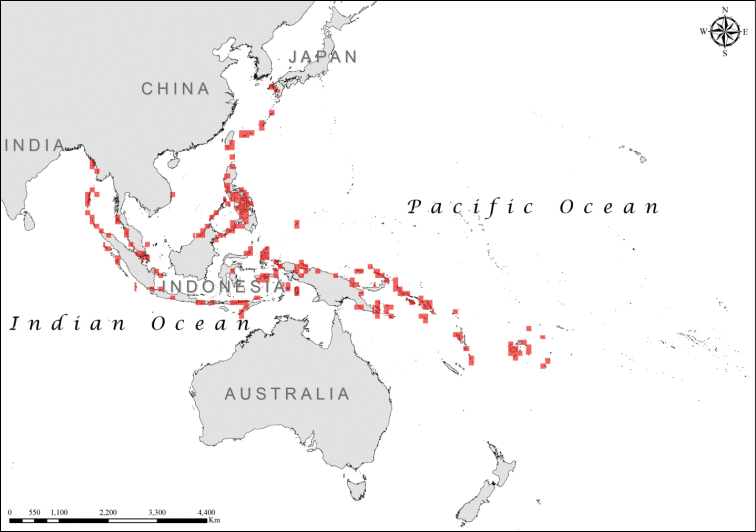
Distribution of *Laticauda colubrina*.

**Figure 4.**
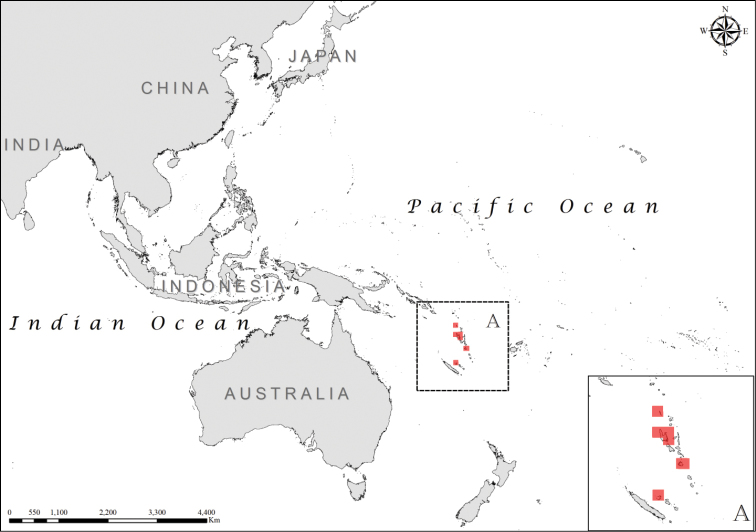
Distribution of *Laticauda frontalis*, regional view and zoomed in (**A**).

**Figure 5.**
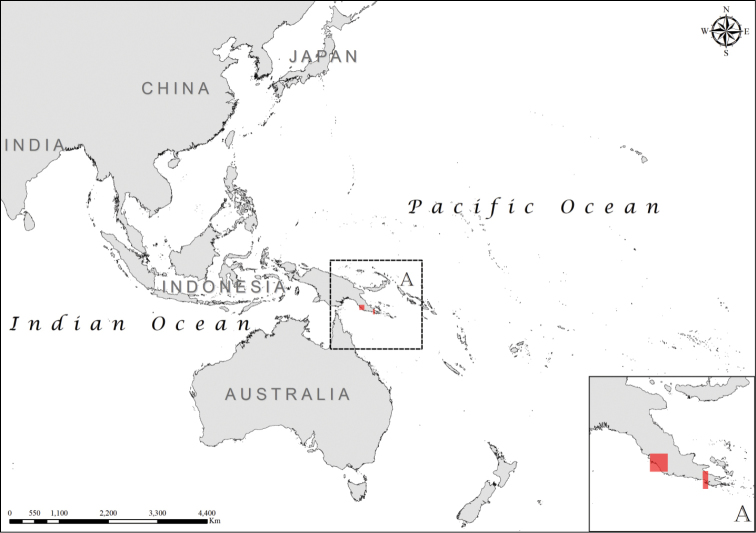
Distribution of *Laticauda guineai*, regional view and zoomed in (**A**).

**Figure 6.**
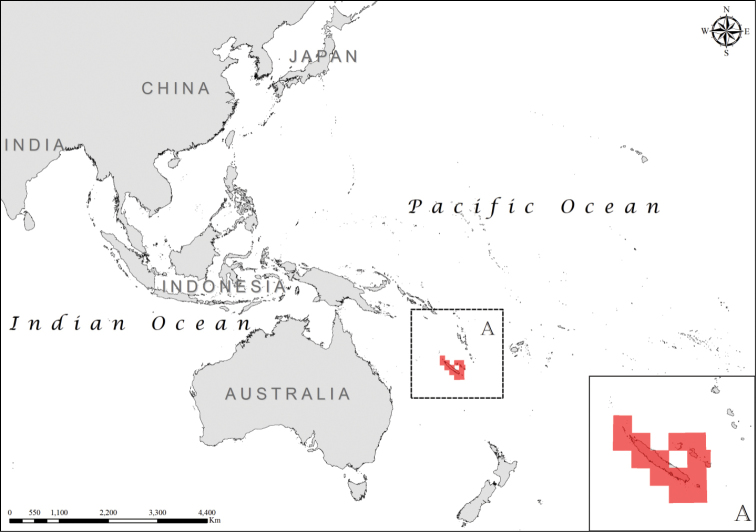
Distribution of *Laticauda saintgironsi*, regional view and zoomed in (**A**).

*Laticauda colubrina* has the largest range of any sea krait species (Table 1; Figure 3; Suppl. material 1), spanning from Tonga, in the south-east, through Fiji, Vanuatu, the Solomon Islands, New Guinea, Palau, most of the Indonesian coast, the Philipines, Taiwan, and reaching its northernmost limits in southern Japan and its westernmost limits in the Bay of Bengal, in the Andaman Islands and on the Myanmar coast (Figure 3; Suppl. material 1). Although the species exhibits a great degree of morphological variability across its distribution range, all populations are currently regarded as a single species (Heatwole 2010; Heatwole et al. 2005; Heatwole and Cogger 2013; Lane and Shine 2011b). In contrast, all other species of the *Laticauda colubrina* group have very narrow distribution ranges. *Laticauda frontalis* is considered endemic to Loyalty Islands and the islands of Vanuatu (Cogger and Heatwole 2006) (Figure 4; Suppl. material 1). Except for this latter location, the available records indicate that the species is sympatric with *Laticauda colubrina* throughout Vanuatu (Figures 3 and 4; Suppl. material 1). *Laticauda guineai* has a very small distribution range, known from only two areas in southern Papua New Guinea (Heatwole et al. 2005) (Figure 5; Suppl. material 1). *Laticauda saintgironsi* is endemic to New Caledonia, including the Loyalty Islands (Figure 6; Suppl. material 1), as reported by other authors (Cogger and Heatwole 2006; Heatwole and Cogger 2013). In the Loyalty Islands, the species occurs in sympatry with *Laticauda frontalis* (Figures 4 and 6; Suppl. material 1).

**Figure 7.**
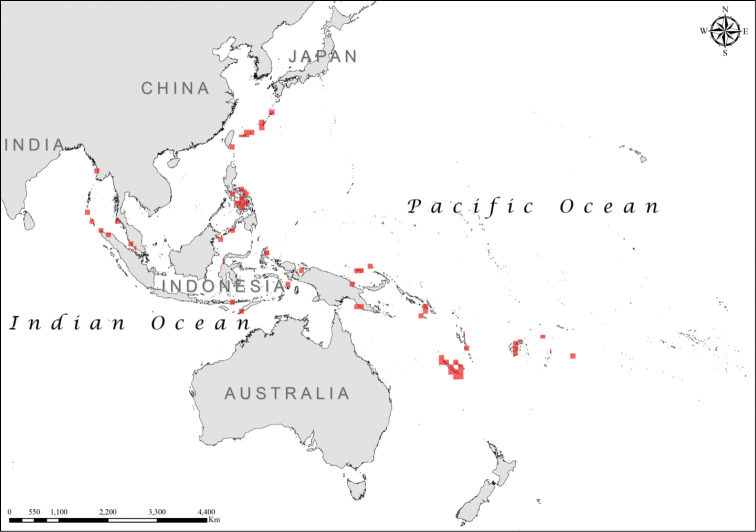
Distribution of *Laticauda laticaudata*.

*Laticauda laticaudata* has a very wide range, similar to that of *Laticauda colubrina* (Figures 3 and 7; Suppl. material 1), but the range of *Laticauda laticaudata* is much more fragmented and the species’ EOO and AOO are considerably smaller (Tables 2–3) than those of *Laticauda colubrina*. The easternmost location of *Laticauda laticaudata* is the Island of Niue while the northernmost and westernmost limits for the species are the same as for *Laticauda colubrina* (Figures 3 and 7; Suppl. material 1). However, *Laticauda laticaudata* also occurs in New Caledonia, where it is sympatric with *Laticauda saintgironsi*, and in Vanuatu, where it is sympatric with *Laticauda frontalis* (Figures 4, 6, and 7; Suppl. material 1). Contrasting with this very broad range, the only other sea krait species from the *Laticauda laticaudata* group, *Laticauda crockeri*, is known from a single location, the Lake Te’Nggano from Rennell Island, Solomon Islands (e.g., Elfes et al. 2013; Heatwole and Cogger 2013). The ecology of this species is virtually unknown (but see Cogger et al. 1987) and the species is considered highly vulnerable (Elfes et al. 2013), thus ecological and conservation research should be prioritized for this species.

**Figure 8.**
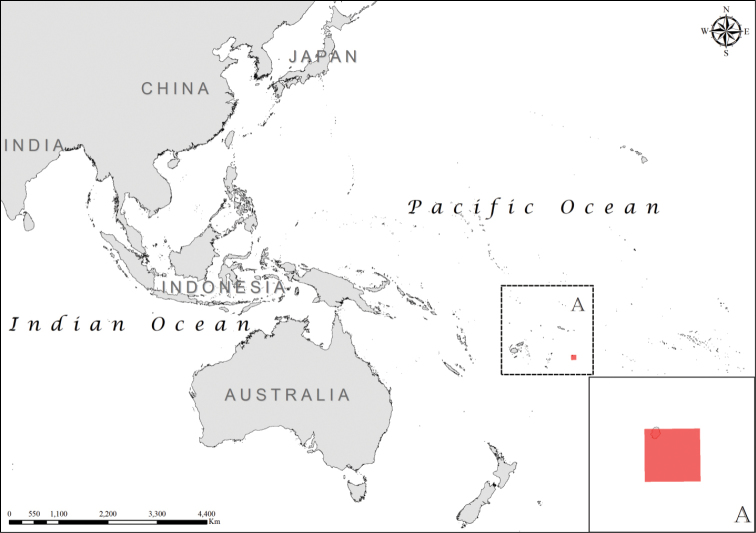
Distribution of *Laticauda schistorhynchus*, regional view and zoomed in (**A**).

**Figure 9.**
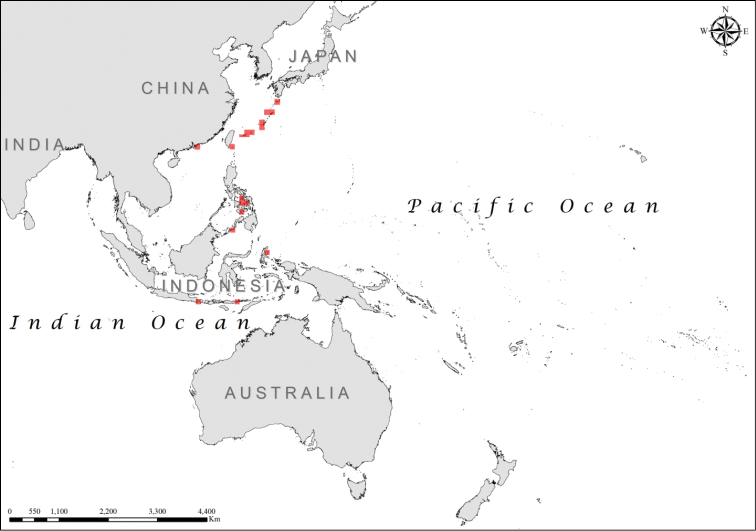
Distribution of *Laticauda semifasciata*.

The *Laticauda semifasciata* group comprises another two species with contrasting and allopatric distribution ranges: *Laticauda schistorhyncha*, endemic to the Island of Niue (Figure 8; Suppl. material 1) (Heatwole and Cogger 2013), and *Laticauda semifasciata*, with a relatively wide but fragmented distribution range, southern Japan being its northernmost limit. Although the southern limit for the species was previously considered to be the Maluku Islands (Heatwole and Cogger 2013), our database indicates an extension of the known range, with new records from southern Indonesia (Figure 9; Suppl. material 1).

**Table 3.** Area of Occupancy (AOO) for the species of *Laticauda* group, calculated based on a 100 km Military Grid (NGA 2014).

Species	Area of occupancy (sq. km.)	Of which	
		% Land	% Ocean
*Laticauda colubrina*	1,988,055.71	22.64	77.36
*Laticauda frontalis*	63,909.17	9.03	90.97
*Laticauda guineai*	13,228.66	69.00	31.00
*Laticauda laticauda*	603,380.15	19.75	80.25
*Laticauda saintgironsi*	152,370.06	13.67	86.33
*Laticauda schistorhynchus*	11,239.95	2.30	97.70
*Laticauda semifasciata*	204,637.92	14.29	85.71

With the exception of one species, all sea kraits presented a terrestrial EOO < 25%. The notable exception is *Laticauda guineai* for which more than 80% of the EOO comprises terrestrial surfaces. In a recent attempt to assess the conservation status of the world’s marine elapids, Elfes et al. (2013) have also calculated values for the extent of occurrence of several *Laticauda* species. The EOO calculated by us for *Laticauda frontalis* (93,874 km^2^) considerably exceeds the value reported by Elfes et al. (2013), which was less than 15,000 km^2^. This discrepancy is due to a coarser grid (NGA 2014) that was used in our study.

For the entire Hydrophiinae, the greatest diversity of hot spots comprise the Gulf of Thailand, the Java Sea, the Timor Sea, Arafura Sea, and the Gulf of Carpentaria (Elfes et al. 2013). Remarkably, the areas of greatest diversity of sea kraits specifically, as identified by the current study, are outside the major hot spots for Hydrophiinae (Elfes et al. 2013), despite broad preferences of most marine Elapids for benthic habitats and coral reefs (Heatwole 1999). This difference suggest that the center of origin for the transition to marine life in Hydrophiinae and in sea kraits may be different, albeit geographically relatively close (Brischoux et al. 2012).

## Supplementary materials

Supplementary material 1 Occurrence dataset of all sea krait species included in this study Data type: Occurrences Explanation note: Geodatabase of occurrence records of all sea krait species compiled in the current study. This dataset is made available under the Open Database License (http://opendatacommons.org/licenses/odbl/1.0/). The Open Database License (ODbL) is a license agreement intended to allow users to freely share, modify, and use this Dataset while maintaining this same freedom for others, provided that the original source and author(s) are credited.Iulian Gherghel, Monica Papeş, François Brischoux, Tiberiu Sahlean, Alexandru Strugariu

## References

[B1] Bonnet X (2012) Long-term field study of sea kraits in New Caledonia: fundamental issues and conservation. Integrative and Comparative Biology 52: 281–295. doi: 10.1093/icb/ics069 22576814 10.1093/icb/ics069

[B2] Bonnet X Brischoux F Pearson D Rivalan P (2009) Beach rock as a keystone habitat for amphibious sea snakes. Environmental Conservation 36: 62–70. doi: 10.1017/S0376892909005451

[B3] Bonnet X Ineich I Shine R (2005) Terrestrial locomotion in sea snakes: the effects of sex and species on cliff-climbing ability in sea kraits (Serpentes, Elapidae, Laticauda). Biological Journal of the Linnean Society 85: 433–441. doi: 10.1111/j.1095-8312.2005.00505.x

[B4] Brischoux F Bonnet X Pinaud D (2009) Fine scale site fidelity in sea kraits: implications for conservation. Biodiversity and Conservation 18: 2473–2481. doi: 10.1007/s10531-009-9602-x

[B5] Brischoux F Bonnet X Shine R (2007) Foraging ecology of sea kraits *Laticauda* spp. in the Neo-Caledonian Lagoon. Marine Ecology Progress Series 350: 145–151. doi: 10.3354/meps07133

[B6] Brischoux F Kato A Ropert-Coudert Y Shine R (2010) Swimming speed variation in amphibious sea snakes (Laticaudinae): A search for underlying mechanisms. Journal of Experimental Marine Biology and Ecology 394: 116–122. doi: 10.1016/j.jembe.2010.08.001

[B7] Brischoux F Shine R (2011) Morphological adaptations to marine life in snakes. Journal of Morphology 272: 566–572. doi: 10.1002/jmor.10933 21337377 10.1002/jmor.10933

[B8] Brischoux F Tingley R Shine R Lillywhite HB (2012) Salinity influences the distribution of marine snakes: implications for evolutionary transitions to marine life. Ecography 35: 994–1003. doi: 10.1111/j.1600-0587.2012.07717.x

[B9] Brischoux F Tingley R Shine R Lillywhite HB (2013) Behavioral and physiological correlates of the geographic distributions of amphibious sea kraits (*Laticauda* spp.). Journal of Sea Research 76: 1–4. doi: 10.1016/j.seares.2012.10.010

[B10] Cogger H Heatwole H Ishikawa Y McCoy M Tamiya N Teruuchi T (1987) The status and natural history of the Rennell Island sea krait, *Laticauda crockeri* (Serpentes: Laticaudidae). Journal of Herpetology 21: 255–266. doi: 10.2307/1563967

[B11] Cogger HG Heatwole HF (2006) *Laticauda frontalis* (de Vis, 1905) and *Laticauda saintgironsi* n. sp from Vanuatu and New Caledonia (Serpentes : Elapidae : Laticaudinae) - a new lineage of sea kraits? Records of the Australian Museum 58: 245–256. doi: 10.3853/j.0067-1975.58.2006.1452

[B12] David P Ineich I (1999) Les serpents venimeux du monde: systématique et répartition. Dumerilia 3: 3–499.

[B13] Elfes CT Livingstone SR Lane A Lukoschek V Sanders KL Courtney AJ Gatus JL Guinea M Lobo AS Milton D (2013) Fascinating and forgotten: the conservation status of the world’s sea snakes. Herpetological Conservation and Biology 8: 37–52.

[B14] Elith J Graham CH Anderson RP Dudik M Ferrier S Guisan A Hijmans RJ Huettmann F Leathwick JR Lehmann A Li J Lohmann LG Loiselle BA Manion G Moritz C Nakamura M Nakazawa Y Overton JM Peterson AT Phillips SJ Richardson K Scachetti-Pereira R Schapire RE Soberon J Williams S Wisz MS Zimmermann NE (2006) Novel methods improve prediction of species’ distributions from occurence data. Ecography 29: 129–151. doi: 10.1111/j.2006.0906-7590.04596.x

[B15] ESRI (2011) ArcGIS Desktop: Release 10.2. Environmental Systems Research Institute, Redlands, CA.

[B16] Ferrier S Watson G (1997) An evaluation of the effectiveness of environmental surrogates and modelling techniques in predicting the distribution of biological diversity. Consultancy report to the Biodiversity Convention and Strategy Section of the Biodiversity Group, Environment Australia. Department of Environment, Sport and Territories, Armidale, Australia.

[B17] Funk VA Richardson KS (2002) Systematic data in biodiversity studies: use it or lose it. Systematic Biology 51: 303–316. doi: 10.1080/10635150252899789 12028734 10.1080/10635150252899789

[B18] Greer AE (1997) The biology and evolution of Australian snakes. S. Beatty.

[B19] Heatwole H (1999) Sea snakes. Krieger Publishing Company, Malabar, 148 pp.

[B20] Heatwole H (2010) Distribution and geographic variation of sea kraits in the *Laticauda colubrina* complex (Serpentes, Elapidae, Laticaudinae). James Cook University.

[B21] Heatwole H Busack S Cogger H (2005) Geographic variation in sea kraits of the *Laticauda colubrina* complex (Serpentes : Elapidae : Hydrophiinae : Laticaudini). Herpetological Monographs, 1–136. doi: 10.1655/0733-1347(2005)019[0001:GVISKO]2.0.CO;2

[B22] Heatwole H Cogger H (2013) Provenance errors and vagrants: their role in underestimating the conservation status of sea fraits (Elapidae: Laticaudinae). Pacific Conservation Biology 19: 295–302.

[B23] IUCN (2012) IUCN Red List Categories and Criteria: Version 3.1. Second Edition IUCN, Gland, Switzerland and Cambridge, UK, iv+32 pp.

[B24] Kharin VE Czeblukov VP (2013) A new revision of sea kraits of family Laticaudidae Cope, 1879 (Serpentes: Colubroidea). Russian Journal of Herpetology 13: 227–241.

[B25] Kidera N Mori A Tu MC (2013) Comparison of freshwater discrimination ability in three species of sea kraits (*Laticauda semifasciata*, *L. laticaudata* and *L. colubrina*). Journal of Comparative Physiology a-Neuroethology Sensory Neural and Behavioral Physiology 199: 191–195. doi: 10.1007/s00359-012-0782-6 10.1007/s00359-012-0782-6 23224248

[B26] Lane A Shine R (2011a) Intraspecific variation in the direction and degree of sex-biased dispersal among sea-snake populations. Molecular Ecology 20: 1870–1876. doi: 10.1111/j.1365-294X.2011.05059.x 21418112 10.1111/j.1365-294X.2011.05059.x

[B27] Lane A Shine R (2011b) Phylogenetic relationships within laticaudine sea snakes (Elapidae). Molecular Phylogenetics and Evolution 59: 567–577. doi: 10.1016/j.ympev.2011.03.005 21414416 10.1016/j.ympev.2011.03.005

[B28] Lillywhite HB Babonis LS Sheehy CM Tu MC (2008) Sea Snakes (*Laticauda* spp.) require fresh drinking water: implication for the distribution and persistence of populations. Physiological and Biochemical Zoology 81: 785–796. doi: 10.1086/588306 18821840 10.1086/588306

[B29] Lillywhite HB Tu MC (2011) Abundance of sea kraits correlates with precipitation. PLoS ONE 6: . doi: 10.1371/journal.pone.0028556 10.1371/journal.pone.0028556 PMC3237450 22194849

[B30] McCarthy CJ (1986) Relationships of the laticaudine sea snakes (Serpentes: Elapidae: Laticaudinae). Bulletin of the British Museum Natural History (Zoology) 50: 127–161.

[B31] MGRS Grid Data layers in GIS Format. http://earth-info.nga.mil [accessed October 17th.2014]

[B32] Pyron RA Burbrink FT Colli GR de Oca ANM Vitt LJ Kuczynski CA Wiens JJ (2011) The phylogeny of advanced snakes (Colubroidea), with discovery of a new subfamily and comparison of support methods for likelihood trees. Molecular Phylogenetics and Evolution 58: 329–342. doi: 10.1016/j.ympev.2010.11.006 21074626 10.1016/j.ympev.2010.11.006

[B33] Roberts JJ Best BD Dunn DC Treml EA Halpin PN (2010) Marine Geospatial Ecology Tools: an integrated framework for ecological geoprocessing with ArcGIS, Python, R, MATLAB, and C++. Environmental Modelling & Software 25: 1197–1207. doi: 10.1016/j.envsoft.2010.03.029

[B34] Rushton SP Ormerod SJ Kerby G (2004) New paradigms for modelling species distributions? Journal of Applied Ecology 41: 193–200. doi: 10.1111/j.0021-8901.2004.00903.x

[B35] Shetty S Shine R (2002) Philopatry and homing behavior of sea snakes (*Laticauda colubrina*) from two adjacent islands in Fiji. Conservation Biology 16: 1422–1426. doi: 10.1046/j.1523-1739.2002.00515.x

[B36] Shine R Bonnet X Cogger HG (2003) Antipredator tactics of amphibious sea-snakes (Serpentes, Laticaudidae). Ethology 109: 533–542. doi: 10.1046/j.1439-0310.2003.00895.x

[B37] Shine R Reed RN Shetty S Cogger HG (2002) Relationships between sexual dimorphism and niche partitioning within a clade of sea-snakes (Laticaudinae). Oecologia 133: 45–53. doi: 10.1007/s00442-002-1012-7 24599368 10.1007/s00442-002-1012-7

[B38] Shine R Shetty S (2001) Moving in two worlds: aquatic and terrestrial locomotion in sea snakes (Laticauda colubrina, Laticaudidae). Journal of Evolutionary Biology 14: 338–346. doi: 10.1046/j.1420-9101.2001.00265.x

